# PFP-GO: Integrating protein sequence, domain and protein-protein interaction information for protein function prediction using ranked GO terms

**DOI:** 10.3389/fgene.2022.969915

**Published:** 2022-09-29

**Authors:** Kaustav Sengupta, Sovan Saha, Anup Kumar Halder, Piyali Chatterjee, Mita Nasipuri, Subhadip Basu, Dariusz Plewczynski

**Affiliations:** ^1^ Laboratory of Functional and Structural Genomics, Center of New Technologies, University of Warsaw, Warsaw, Poland; ^2^ Department of Computer Science and Engineering, Jadavpur University, Kolkata, India; ^3^ Laboratory of Bioinformatics and Computational Genomics, Faculty of Mathematics and Information Science, Warsaw University of Technology, Warsaw, Poland; ^4^ Department of Computer Science and Engineering, Institute of Engineering and Management, Kolkata, West Bengal, India; ^5^ Department of Computer Science and Engineering, Netaji Subhash Engineering College, Kolkata, India

**Keywords:** protein sequence, protein domain, protein-protein interaction network, 3D gene-gene association, ranked GO, protein function prediction

## Abstract

Protein function prediction is gradually emerging as an essential field in biological and computational studies. Though the latter has clinched a significant footprint, it has been observed that the application of computational information gathered from multiple sources has more significant influence than the one derived from a single source. Considering this fact, a methodology, PFP-GO, is proposed where heterogeneous sources like Protein Sequence, Protein Domain, and Protein-Protein Interaction Network have been processed separately for ranking each individual functional GO term. Based on this ranking, GO terms are propagated to the target proteins. While Protein sequence enriches the sequence-based information, Protein Domain and Protein-Protein Interaction Networks embed structural/functional and topological based information, respectively, during the phase of GO ranking. Performance analysis of PFP-GO is also based on Precision, Recall, and F-Score. The same was found to perform reasonably better when compared to the other existing state-of-art. PFP-GO has achieved an overall Precision, Recall, and F-Score of 0.67, 0.58, and 0.62, respectively. Furthermore, we check some of the top-ranked GO terms predicted by PFP-GO through multilayer network propagation that affect the 3D structure of the genome. The complete source code of PFP-GO is freely available at https://sites.google.com/view/pfp-go/.

## Introduction

In recent years, protein function prediction has started using integrated function predictive information from several sources (Piovesan et al., 2015) instead of using a single source of information. These include Protein-Protein Interaction (PPI), Protein domain, Amino Acid Sequence of protein, Protein’s structure, Genomic information, etc. Though integrated information enriched classifiers should perform better than a single type of feature, the design of such a single classification system of heterogeneous data sources is challenging for the Proteomic research community. Moreover, the prediction task becomes more challenging as it is characterized by several factors: 1) Any protein may be associated with multiple functions, i.e., one-to-many relationships, 2) functional groups are numerous, and 3) their existence is hierarchically structured and unbalanced.

The functionality of a protein can be attributed to the physical interactions represented in Protein-Protein Interactions (PPIs). Using proximity relationships between connected proteins, computational methods attempt to propagate the labels of known proteins to unknown proteins across the network. PPIs are mediated by their constituent domain-domain interactions (Chatterjee et al., 2011). Genes evolved from the same ancestral gene are functionally similar. So, finding known genes with sufficient sequence similarity is a powerful way to predict function. These various types of data individually are not sufficient to annotate functional groups.

Moreover, a recent trend shows that hierarchical relationships between functional classes motivate the development of hierarchy-aware prediction methods, which are significantly better than hierarchy-unaware “flat” prediction methods (Pandey et al., 2006). Functional relationships, e.g., Taxonomy like Gene Ontology (GO) (Ashburner et al., 2006) or FunCat (Ruepp et al., 2004), can be exploited to improve the predictive performance of learning algorithms.

Motivated by the facts mentioned above, here we propose an integrated approach where their orthogonal methods, namely, constituent domains of the protein and their interactions, sequence homology, and protein interaction data, are used to assign functional GO terms for unknown proteins and then these prediction decisions are combined into a consensus decision using n-star approach and functional enrichment. The following section discusses the current state of the arts of computational techniques in the protein function prediction domain based on raw amino sequence, domain, and protein interaction data.

### Sequence-based approaches for protein function prediction

Genes that evolved through duplication and rearrangement from single ancestral genes are known to be homologous. The homologous genes are found at various places in the same genome. However, duplication is considered paralogous, whereas some orthologous genes diverge through speciation events found in different organisms. Inference of functional terms from sequence similarity is well supported by Anfinsen’s dogma claiming protein’s sequence determines protein’s tertiary structure (Anfinsen, 1973). The basic strategy of the sequence-based prediction method is that similar known proteins are searched from a database for any target protein, assigning associated GO terms to that protein of interest. Local alignment-based tools like SSEARCH (Pearson, 1995) take a target sequence and find top hit sequences along with their statistically significant score E-value with the Smith-Waterman dynamic programming algorithm. As it is time-consuming, FASTA (Pearson and Lipman, 1988) does pairwise alignments only on highly similar regions using a lookup table, and BLAST (Altschul et al., 1990) saves time with the use of pre-computed similar words. However, these strategies are not sensitive to all protein families with different conservation degrees. On the other hand, PSI-BLAST (Altschul et al., 1997) uses sequence profile instead of raw sequence. It makes a profile of the target sequence and similar sequence at each iteration and uses a computed profile at the next iteration.

Pre-computed profiles of protein domains or portions of the conserved region can be used for assigned tasks. BLOCKS (Pietrokovski et al., 1996), ProDom (Corpet et al., 2000), Pfam (Finn et al., 2016), and SUPERFAMILY (Pandit et al., 2002) are datasets of profiles of protein domains, PRINTS (Attwood, 2002) is collection of protein fingerprints. Here target sequence and similar sequences are represented in the profile where the target protein profile is matched against the database sequence profile.

Sequence-based prediction is easy to use because most proteins are available with their sequence and functional annotation. However, limitations to sequence-based methods arise when 60% of the sequences are similar (Kihara, 2011). A correlation between structural and functional similarity can be used in that case. Sequence-based prediction methods are helpful when only raw protein sequence information is available. However, sometimes it becomes challenging because wrong functional annotation may be propagated for functional assignment, or correct function prediction does not always take place as important issues are not always considered, like non-orthologous displacement of genes or proteins has multiple domains (Chitale et al., 2009; Halder et al., 2019).

### Domain-based approaches for protein function prediction

Protein domains are independent units of protein function which have unique three-dimensional structures. Protein functions are collective results of functions of its constituent domains. So, to predict function, exploiting the domain architecture of proteins is the need of the hour and their interaction and cooperation pattern. Some current *state-of-the-art* techniques use domain information for function prediction. Peng et al. (2014) use protein domain information along with Protein interaction networks and complexes. The domain combination similarity (DCS) representing the domain compositions of both proteins and their neighbors is used in their algorithm. Robert Rentzsch and Christine A Orengo (Rentzsch and Orengo, 2013) derive a functional family by combining sequence clustering with supervised cluster evaluation.

INGA (Piovesan et al., 2015) is another predictor using domain architecture and transfer annotation from proteins sharing the same domain pattern. It identifies putative PFAM domains, and all proteins associated with GO terms from UniProt are retrieved, and finally, those GO terms are assigned to the target protein (Piovesan et al., 2015).

### Protein interaction network-based approaches for prediction of protein function

Protein interactions have great importance in protein function, so the function of an unknown protein can be extrapolated from the functional annotation of its interaction profiles exploiting proximity relationships. There are mainly four categories of network-based approaches in functional inferences of protein: Markov random field based, optimization-based, Clustering based, and neighbor based (Pandey et al., 2006). The trend also combines decisions about protein functions obtained through different approaches. Neighborhood-based approaches are primarily based on the idea that the proteins closer to the network are more similar in functionality. In prior studies (Schwikowski et al., 2000; Hishigaki et al., 2001), the functionality of target proteins is assigned considering the probability of occurrence of functions among the neighboring proteins. However, these approaches limited the neighboring proteins to level-1 of the target proteins in assigning the functional annotations. In another study (Chen et al., 2007) of protein function, further advancement has been introduced in the neighbor-based approach by introducing the network motifs concept in protein interactome. A global optimization mechanism was introduced in functional assignments to its unclassified (target) partners in the PPIN (Vazquez et al., 2003). In other work (Karaoz et al., 2004), the functional linkage graphs have been mapped into a variant of a discrete-state Hopfield network in order to gain the maximally consistent assignment by minimizing an “energy” function that includes a heuristic-guided local search mechanism. However, Karaoz et al. (2004) focused more on the global properties of interaction maps but not on the local proximity of interacting proteins (Nabieva et al., 2005). To overcome these above-described issues and considering the distant effects of annotated proteins, Nabieva et al. (2005) have introduced a Functional Flow based strategy using network flow where each protein of known function is annotated as a “source” of “functional flow.” The work of Deng, Mehta et al. (2002) and Letovsky and Kasif (2003) are worth mentioning among probabilistic approaches. Functional module detection (Bader and Hogue, 2003; Sharan et al., 2007) and graph clustering methods (Spirin and Mirny, 2003; King et al., 2004) are effective module-assisted approaches.

### Integrated approaches based on sequence, domain, and protein-protein interaction networks for protein function prediction

Inference of functional terms from sequence similarity is well supported by Anfinsen’s dogma claiming protein’s sequence determines protein’s tertiary structure (Anfinsen, 1973). The basic strategy of the sequence-based prediction method is that for any target protein, similar known proteins are searched from a database using sequence similarity tools, like, PSI-BLAST, and assign those associated GO terms to that protein of interest. Local alignment-like tools take top hit sequences with a significant E-value or Smith-Waterman alignment score. A global or local alignment algorithm is used to infer homology (Altschul et al., 1997). DNA binding site prediction is also considered to be sequence-based function prediction. Sequence information and various types of sequence-derived features, Physico-chemical properties (for example, polarity, hydrophobicity), PSSM, the composition of amino acid, dipeptide composition, structural features like secondary structure, solvent accessible surface area are used (Altschul et al., 1997; Halder et al., 2019; Pearson, 1995; Pearson & Sierk, 2005). Protein subcellular location prediction (Garg et al., 2005; Sarda et al., 2005), enzyme function prediction (Wang et al., 2010), and signal peptide prediction (Nielsen et al., 1997) can also facilitate the prediction of protein function. The use of machine learning algorithms (Wang and Xiao, 2014; Xiao et al., 2012) and nearest neighbor classifier (Huang and Li, 2004; Wang and Yang, 2010) is significant in this regard. Sequence-based prediction is easy to use because most proteins are available with their sequence and functional annotation. However, limitations to sequence-based methods arise if two sequences have similarities below 60% (Kihara, 2011). A correlation between structural and functional similarity can be used in that case.

Prediction of protein function can also be performed by domain information of protein. Protein domains are independent folding units that represent basic functional units of protein. In another work (Rentzsch and Orengo, 2013), the function prediction is made using domain families derived through sequence clustering. Forslund and Sonnhammer (2008) computationally assign GO terms to unknown proteins based on the presence of identifiable domains. Rule-based and probabilistic models investigate the dependence between protein domain content and function.

Proteins perform their functions through interaction with each other. Moreover, a protein is associated with multiple functions. So, inference of function for any unknown protein can be made from its interaction information with its interacting partner. Recently, computational function prediction techniques are gaining importance from PPIN also. Network-based approaches are classified into the following groups: neighbor-based (Saha et al., 2014), optimization-based (Chen and Xu, 2004; Deng, Sun, et al., 2002) (Schwikowski et al., 2000), Markov random field based (Deng, Sun, et al., 2002) and Clustering based (Dandekar et al., 1998).

Some of the other relevant works of protein function prediction have been summarized in Table 1. Besides, some advanced studies use multi-features obtained from protein and sequence (Bao et al., 2017, Bao et al., 2021, Bao et al., 2022). Considering all these existing works, it is observed that there is still a pressing need to explore specific areas where heterogeneous sources are blended for a common cause of predicting protein function. This work proposes a methodology named PFP-GO to capture the protein functional dependencies on the domain, sequence, and PPI. The main idea is to embed all available information sources to consider other essential features rather than using sequence, domain, or PPI alone. All the source code of PFP-GO is available on https://sites.google.com/view/pfp-go/.

**TABLE 1:** Current computational methodologies of protein function prediction.

Features used	Brief description	References
Sequence and Network	A deep learning framework for gene ontology annotations with sequence- and network-based information	F. Zhang et al., (2020)
	DeepFunc: A deep learning framework for accurate prediction of protein functions from protein sequences and interactions	F. Zhang et al., (2019)
	Predicting GO annotations from protein sequences and interactions	X. Zhang et al., (2021)
GO terms	A deep learning framework for predicting protein functions with co-occurrence of GO terms	M. Li et al., (2022)
	Gene function prediction based on gene ontology hierarchy preserving hashing	Zhao et al. (2019)
	Gene function prediction based on combining gene ontology hierarchy with multi-instance multi-label learning	Z. Li et al., (2018)
Structure	Structure-based protein function prediction using graph convolutional networks	Gligorijević et al. (2021)
	Structure-based function prediction: approaches and applications	Gherardini and Helmer-Citterich, (2008)
	prediction of protein function from structure: insights from methods for the detection of local structural similarities	Najmanovich et al. (2005)

## Methodology

PFP-GO assigns functional groups to target proteins based on the information integration of sequence similarity, PPI networks, and domain assignments. This method combines these orthogonal predictions and derives consensus predictions for GO terms using functional enrichment. As different heterogeneous information sources are used, mapping data from one source to another is essential in this regard. Protein interaction networks may contain specific false positive and false negative data. So, finding biologically essential proteins is challenging as they are promising candidates for finding drug targets. This work categorizes PFP-GO into four sections: 1) It identifies the functionally active target proteins (i.e., proteins associated with frequently occurring GO terms) whose functional groups are predicted. 2) Each target protein’s level-2 neighborhood graph is considered, and non-essential proteins, i.e., shore, bridge, and fjord proteins (Hanna and Zaki, 2014), are eradicated. 3) Once the refined PPI for each target protein is obtained, sequence-based, domain-based, and neighborhood protein interaction-based approaches are applied to the target and its neighbors to assign GO terms. 4) GO terms are ranked using a functional enrichment score. 5) Finally, common GO terms among the sequence-based, domain-based, and neighborhood protein interaction-based prediction results are finally transmitted to the target protein following a 3-star consensus (Chatterjee et al., 2016) approach.

### Database

PFP-GO can be centrally categorized into three sections: 1) Sequence-based prediction, 2) Domain-Domain interaction-based prediction, and 3) Topology or neighborhood-based prediction from the PPI network. In topology or neighborhood-based prediction from PPI networks, String (Franceschini et al., 2013) and Uniprot (Consortium, 2015) databases are used to generate PPI network and GO terms, respectively. In domain-domain interaction-based prediction, PFAM (Finn et al., 2016) and DOMINE database (Yellaboina et al., 2011) are used for obtaining the domain-domain interaction from the corresponding Uniprot ids. GO Consortium database (Consortium, 2018) also plays an essential role in this section in including its own GO terms. While in sequence-based prediction, STRING and Uniprot are utilized in coordination for protein sequence and GO term generation.

### Identification of functionally active targets

**FIGURE 1:**
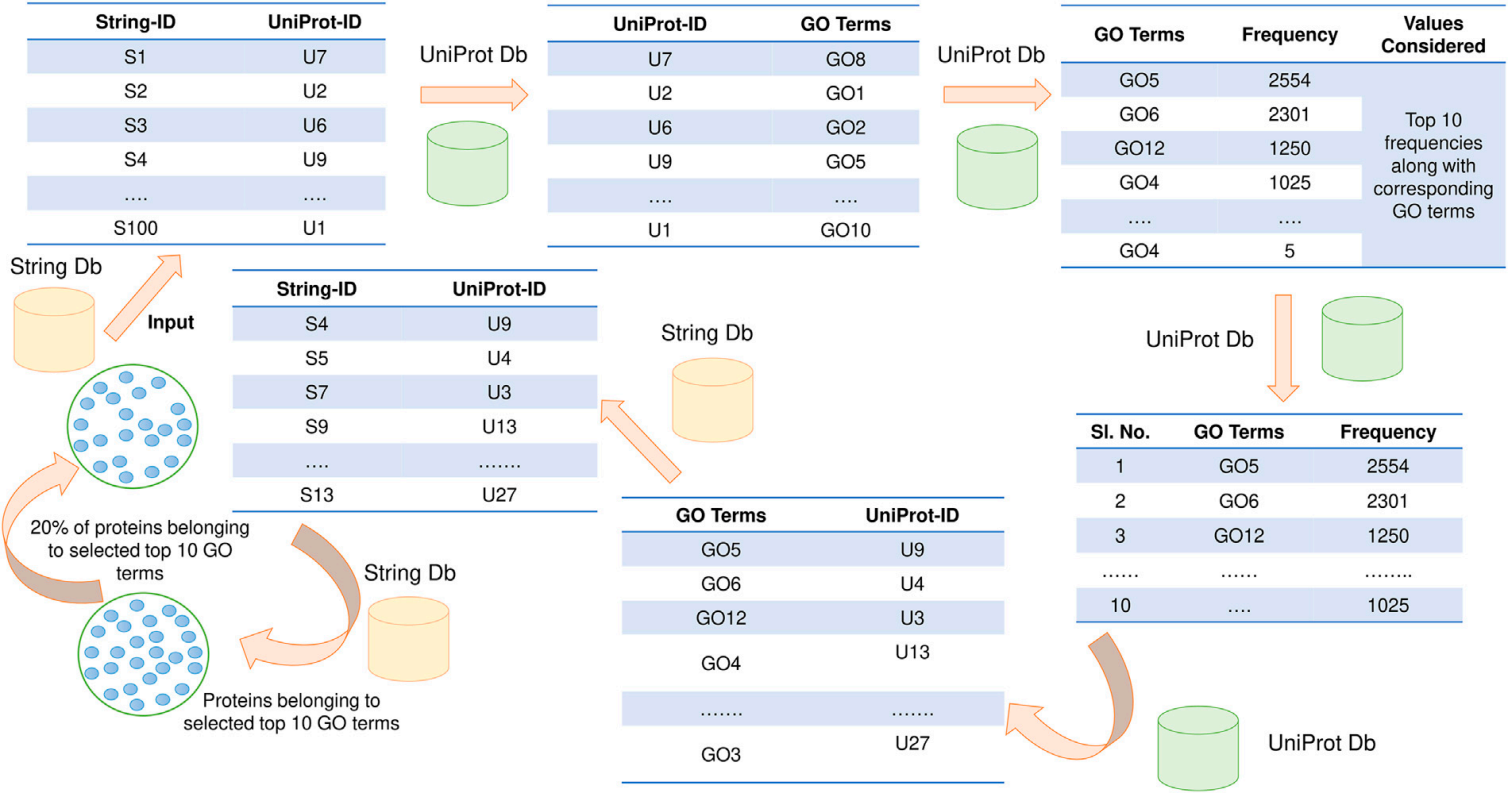
Functionally active target protein selection. Two databases: STRING and UniProt, have been used for this purpose.

In this section, the STRING database id used to fetch interactions (Franceschini et al., 2013), and the UniProt database was used for GO annotations (U. Consortium, 2015). If direct mapping from STRING ID to UniProt ID is unavailable, then PFP-GO focuses on identifying a homogenous entry with at least 90% sequence identity from UniProt. Next, computation of the frequency of associated GO terms for every protein is implemented. The top 10 frequently occurring GO terms are considered based on the frequency of GO terms. The STRING IDs are fetched from these corresponding GO terms using UniProt as an intermediary. 20% of these proteins (STRING IDs) are randomly considered to be functionally active target proteins. The schematic diagram of selecting active target proteins is highlighted in Figure 1.

### Pruning and filtering of target protein neighborhood graph

**FIGURE 2:**
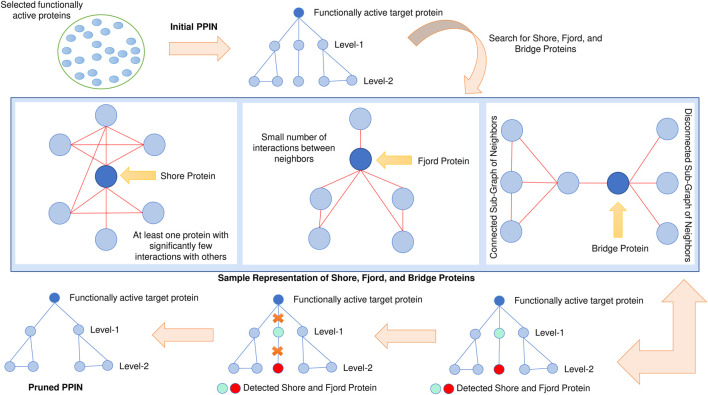
Pruning of target neighborhood graphs. Bridge, fjord, and shore proteins are detected and pruned.

For each target protein, interaction information is retrieved from the STRING database (Franceschini et al., 2013), and their neighborhood graph (consisting of level-1 and level-2 proteins) is formed. To remove non-essential protein, a topology-based method is considered for testing the target protein neighbor’s essentiality. In order to assess the essentiality, it is checked whether any protein in the target’s neighborhood is of bridge or Fjord or shore protein (Hanna and Zaki, 2014). These neighbors get ultimately pruned to ensure that their presence might not affect the prediction accuracy (see Figure 2).

**FIGURE 3:**
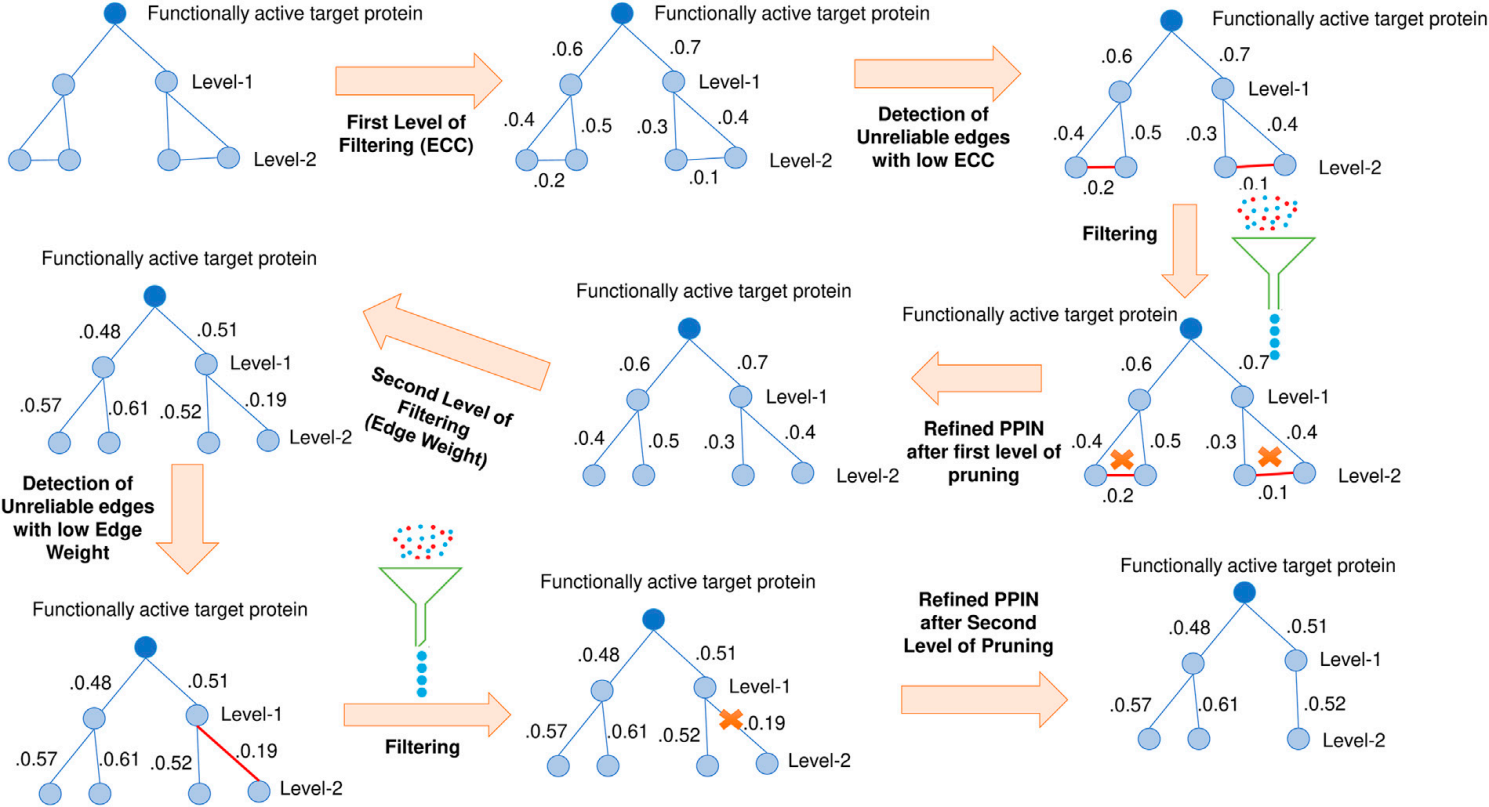
Double filtering of target neighborhood graph. Edge clustering coefficient and edge weight are computed based on which non-essential nodes are filtered.

Besides this bridge, fjord and shore proteins, Network centrality-based Edge Clustering Coefficient (ECC) (Peng et al., 2012), and edge-weight (S. Wang and Wu, 2013) are yet another two most effective measures for the identification of essential proteins. ECC measures the degree of closeness between two nodes in a graph. Those edges with higher ECC values are more likely to be in a module. In comparison, edge-weight assigns a weightage to each edge, which signifies the reliability of the corresponding edge. So double filtering using both ECC and edge-weight is also implemented on the pruned target neighborhood network to ensure the presence of the most reliable edges. The schematic diagram of this 2-pass filtering approach has been highlighted in Figure 3. The threshold of both ECC and edge-weight is calculated by the following mathematical equation (Zhang et al., 2016):

$$Threshold=\alpha +k\times \sigma \times \left(1-\frac{1}{1+\sigma^{2}}\;\right)$$

where k∈{3} defines high cut-offs. α and σ are considered to be the mean and standard deviation of ECC/edge weight values. Once the target neighborhood network is refined by double filtering, functions of target proteins are predicted using protein sequence, protein domain, and PPI network separately, which will be discussed in the upcoming sections.

### Sequence-based prediction

In this section, the functions of target proteins are predicted using protein sequences. Since proteins are formed of amino acids, protein sequence always plays a significant role in target protein function prediction. Sequence-based information is extracted by computing and assigning a Physico-chemical property score to all proteins in the target neighborhood graph, including the target itself. Physico-chemical property score (Jiang and McQuay, 2011) is the average of the values obtained from various Physico-chemical properties of protein/amino acid sequence. In this proposed work, seven Physico-chemical properties are considered, which are:

• *Extinction Coefficient (Eprotein)*
• *Absorbance (Optical Density)*
• *Number of Negatively Charged Residues (Nneg)*
• *Number of Positively Charged Residues (Npos)*
• *Aliphatic Index (AI)*
• *IP/mol weight*
• *Hydrophobicity (Hphb)*

Initially, node degree is computed for each member belonging to the refined neighborhood network for each target protein. The node degrees are then sorted in descending order. Now protein clusters are formed for each target protein. The protein with the highest node degree is selected as the first seed of the cluster. Then the distance between the seed and other proteins in the neighborhood of each target protein is computed based on the Euclidean distance. The Physico-chemical property score serves as an input to the Euclidean distance. If the distance is less than a specific threshold, then the inter-connected protein of the seed is incorporated in the cluster and is removed from the node degree list. Then the next node with the highest degree is considered a seed, and its corresponding clusters are formed similarly to the previous one. Thus, the clusters created are validated using inter-cluster and intra-cluster distances so that no miss classification or overlap is present.

**FIGURE 4:**
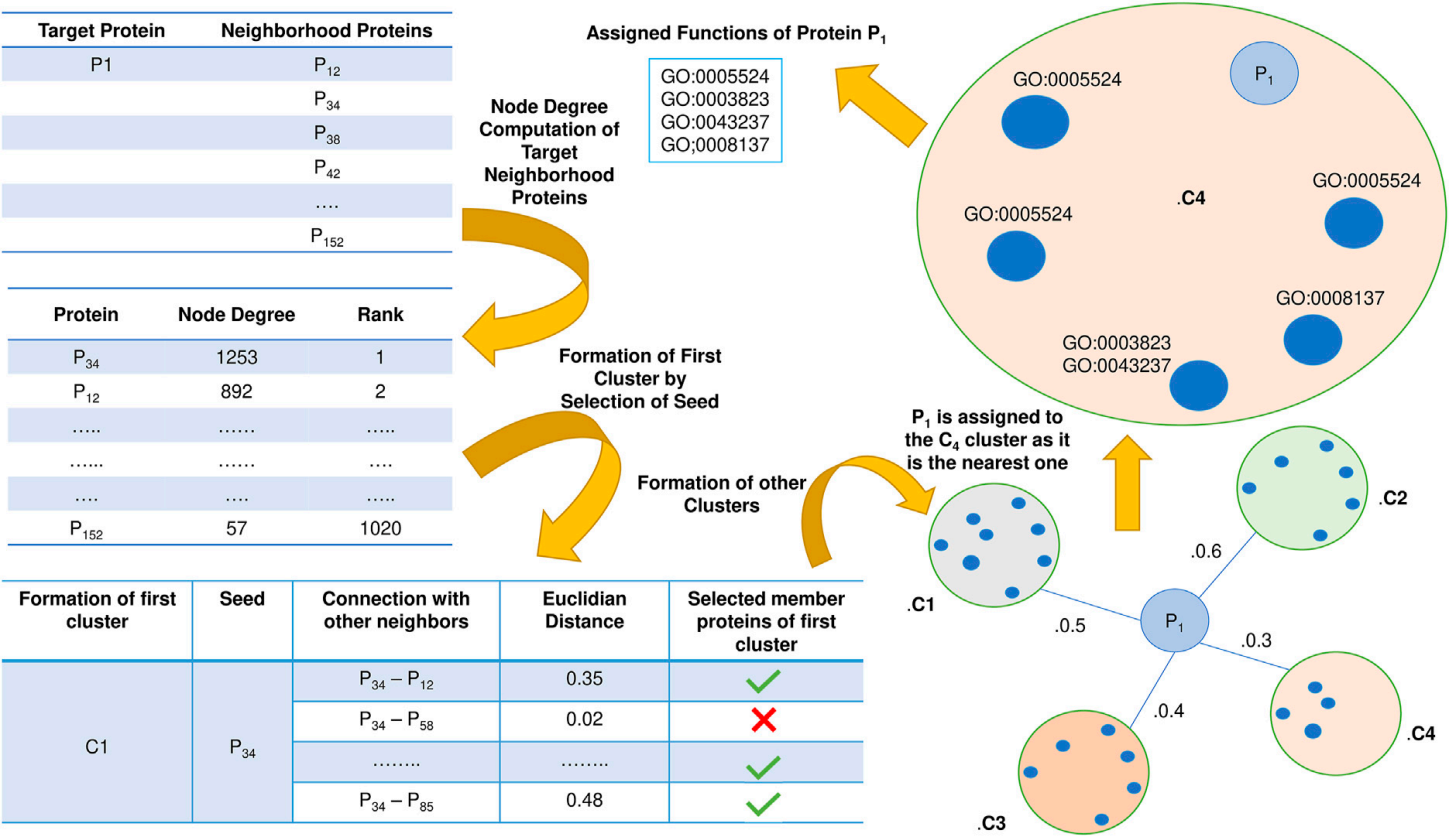
Schematic representation of Sequence-based protein function prediction. The essential aspects of this phase are the selection of seeds followed by the formation of clusters and computation of Physico-chemical properties.

Once the cluster formation in each target protein’s neighborhood is finished, each cluster’s Physico-chemical property score is evaluated. Physico-chemical property score of a cluster is nothing but the average of the earlier computed Physico-chemical score of each constituent protein in the corresponding cluster. Then the Euclidean distance of the Physico-chemical property score between each target protein and its corresponding neighborhood clusters is calculated, and the target protein is assigned to the nearest cluster. Now the cluster contains more than one protein. So, it is not desirable that the functions of all the existing proteins in the chosen cluster are assigned to the target since it will enhance the false positives leading to an abrupt fall in the prediction accuracy level. Considering this fact, intra-cluster distance based on the Euclidean distance of the Physico-chemical property score is computed between the target and the other remaining proteins in the corresponding cluster. Functions of the selected protein having the least distance are assigned to the target protein. The schematic diagram of the entire sequence-based prediction is shown in Figure 4.

### Domain-domain interaction-based prediction

**FIGURE 5:**
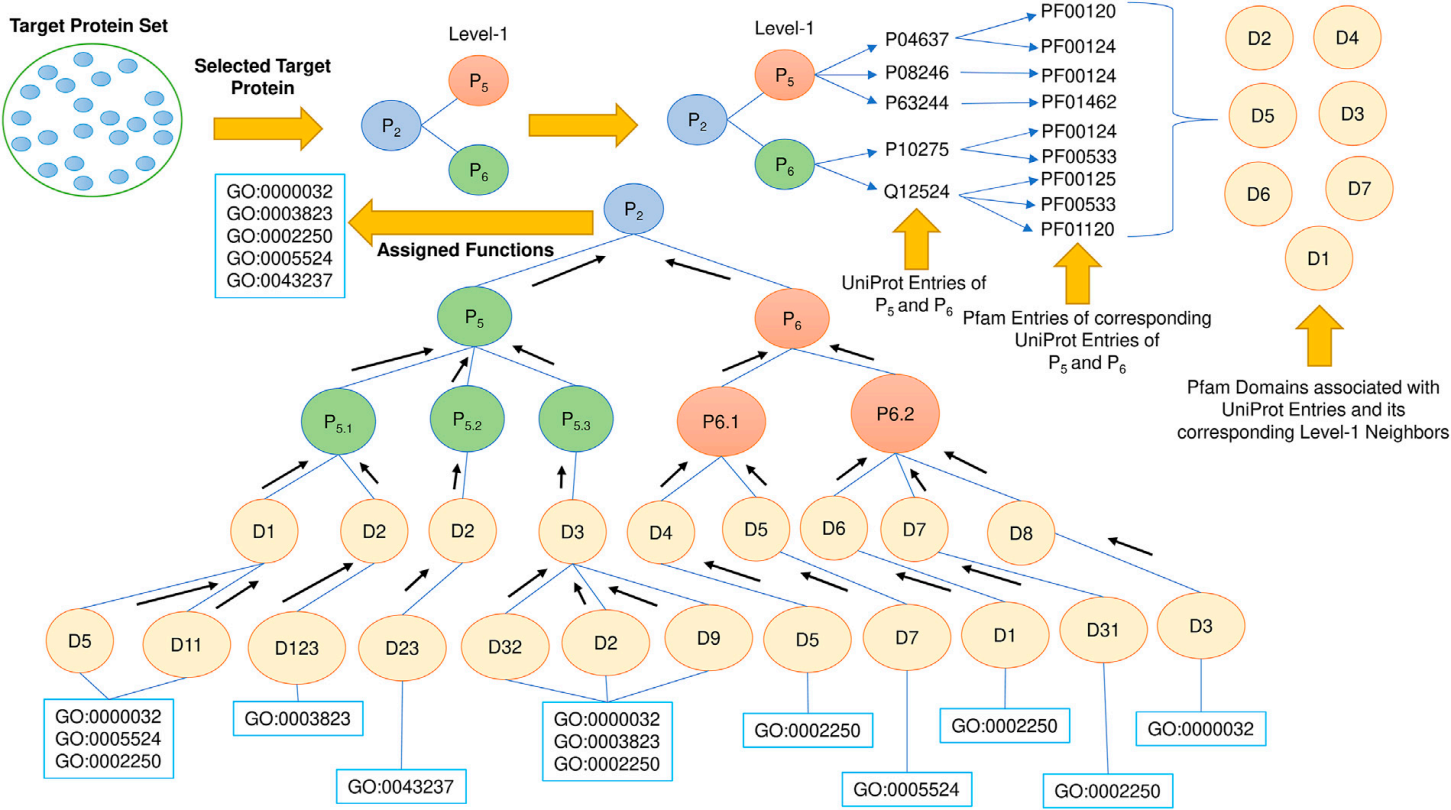
Working strategy of Domain-based protein function prediction. Four databases, String, UniProt, PFAM, and DOMINE, are used in this phase.

Protein domains are the independent units that are responsible for protein function. The study of domain-domain interaction may lead to better protein function prediction. So, this proposed methodology uses PFP-GO protein domains for target protein function prediction. For each node in the refined neighborhood graph of the target protein, its STRING-id is fetched from the STRING database. Each of these STRING-ids is mapped to the Uniport database to obtain its corresponding Uniprot-id. These Uniprot-ids are mapped to their respective PFAM entries (ids), which are used to fetch the PFAM domains using the PFAM database. These derived PFAM domains of the neighborhood graph of each target protein are also checked and validated using the DOMINE database. All the mappings are considered if the one-to-many mapping occurs during the linking as mentioned earlier between databases. Once all the PFAM domains are obtained, the GO terms (protein functions) corresponding to these interacting domains are deduced from the GO Consortium database. Each of these GO terms is assigned a particular ranking based on the frequency of their occurrence. GO terms with the highest ranking are back propagated from the bottom to the top and allocated to the target protein. The entire schematic diagram for this phase has been highlighted in Figure 5.

### Topology or neighborhood-based prediction from protein-protein interaction network

**FIGURE 6:**
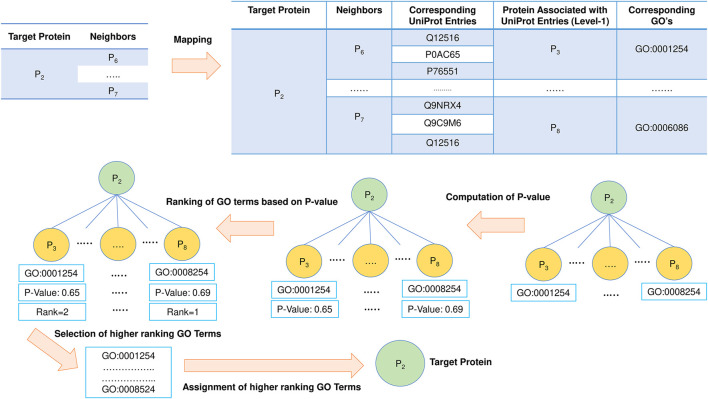
PPI network-based protein function prediction. GO term enrichment with *p* value is vital in this phase.

The functionality of a protein is governed by its topological position in the network. The proteins in the densely related subgraph in the PPI network tend to be more significant in disease propagation and drug targets. This is the most important aspect for which the neighborhood graph of each target protein in PFP-GO gets pruned and filtered before proceeding with the function prediction of the target proteins. In this section, at first, Uniprot-id from Uniprot are fetched from the corresponding STRING-id of each level-1 protein in the pruned neighborhood graph of the target protein. The associated GO terms and proteins (considered level-1 of the target) with these Uniprot-ids are derived from the Uniprot database. The same is also implemented for level-2 of the target protein (Sengupta et al., 2018). Once all the GO terms are fetched, each of them is ranked by computing its enrichment in the PPI network using the *p* value with Fisher’s exact test (Piovesan et al., 2015). Top-ranked GO terms are finally allocated to the target protein. The schematic diagram for this phase of PPI network-topology-based prediction is shown in Figure 6.

### Integrated prediction using sequence, domain, and protein-protein interaction

The consensus technique is more effective if the results from orthogonal sources are merged to generate consistent results. In PFP-GO, PPI network, domain, and sequence data are used. For the final prediction, the n-star consensus method is used. As discussed earlier, the proposed method uses a 3-star consensus between three different predictors. 1-star consensus assigns those GO terms predicted by at least one of the predictors to the target protein. The 1-star is the least reliable as it gives the maximum number of GO terms. The 2-star consensus assigns the GO terms commonly predicted by at least two different predictors to the target protein. In contrast, the 3-star consensus (Chatterjee et al., 2016) is the most reliable as it only assigns GO terms commonly predicted by all three predictors for the target protein.

## Results

**FIGURE 7:**
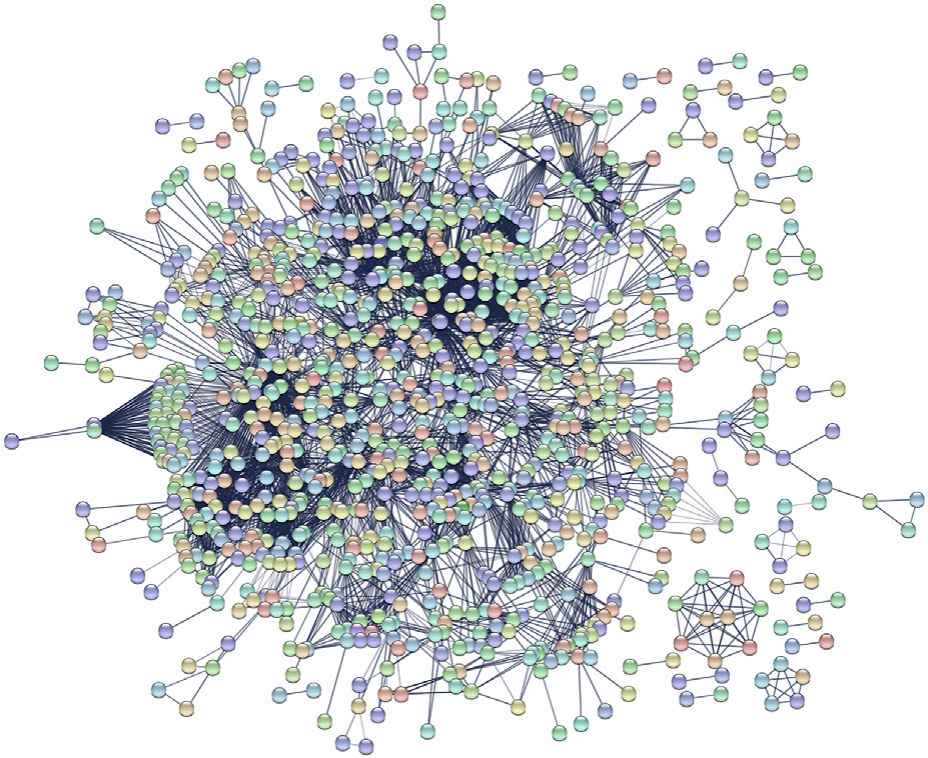
Sample human PPI network from STRING database. It consists of nodes and interactions between them.

PFP-GO first selects a functionally active protein set from the database, considered target proteins. It then predicts the functions of the target proteins using sequence-based, domain-based, and neighborhood Protein Interaction based approaches discussed earlier in the methodology section. The proposed method fetches the proteins in the String database for the target set selection. These proteins are mapped to the UniProt database. The STRING database contains 19,247 human proteins mapped to the UniProt database to retrieve the associated GO terms. PFP-GO utilizes the mapping of UniProt to retrieve GO terms because String database entries are not directly associated with GO annotation. In this proposed work, the human PPI network of String databases is used because it has 85, 58,002 interactions which is significantly higher than UniProt, which has only 46,410 interactions. A network diagram of the Human String database consisting of 2000 nodes is highlighted in Figure 7. Since the mapping between String and UniProt is one-to-many, each string entry has one or more UniProt IDs. However, suppose the mapping is not present for a particular protein. In that case, the corresponding sequence of String data is fetched, and the proteins having 90% similarity with it are considered from the UniProt database. Their corresponding GO terms are also used for further experimentation. In the UniProt database, out of 12,366 GO terms, 1547 GO terms fall under the Cellular Components category, while 4,105 and 11,263 GO terms are classified under Molecular Function and Biological Process, respectively. Then the frequency of each GO term is calculated, and the top 10 GO terms are fetched along with their UniProt IDs. PFP-GO detects 9,141 proteins associated with the top 10 GO terms, which is reverse mapped to the STRING dataset to get 6,999 proteins. Then it selects a random 20% of proteins out of 6,999, which is near about 1,400 proteins. From these 1,400 proteins, 639 unique proteins are finally filtered out after redundancy removal. These proteins are ultimately considered functionally active targets.

The performance measure of PFP-GO is evaluated using Precision (P), Recall (R), and F-Score (F). PFP-GO is a combined methodology based on three heterogeneous resources, i.e., protein sequence, protein domain, and PPI network. It achieves an overall precision, recall, and F-score of 0.67, 0.58, and 0.62, respectively. It is initially compared with INGA (Piovesan et al., 2015), as shown in Table 2, since it uses the same heterogeneous resources as that PFP-GO. However, INGA lacks proper filtering and pruning of the neighborhood graph of the target protein. Besides consideration of Physico-chemical properties of protein sequence in PFP-GO instead of just applying sequence comparison algorithm to estimate sequence similarity is another aspect for outperforming INGA.

**TABLE 2:** Performance Analysis of INGA and PFP-GO based on PPI network, sequence, and domain.

Methodology	Precision	Recall	F-score
PFP-GO	0.67	0.58	0.62
INGA Piovesan et al., (2015)	0.44	0.51	0.47

The same has also been compared with four of the existing methods: FunApriori (Prasad et al., 2017), the neighborhood counting method (Schwikowski et al., 2000), the chi-square method (Hishigaki et al., 2001), a recent version of the neighbor relativity coefficient (NRC) (Moosavi et al., 2013), FS-weight based method (Chua et al., 2006). It should be noted here that all these methods are based on the PPI network alone. To remove biases and to compare in a common field, performance analysis of PFP-GO is estimated on the prediction of the PPI network only. The performance is highlighted in Table 3.

**TABLE 3:** Performance analysis of PFP-GO with other methods based on PPI network.

Methodology	Precision	Recall	F-score
PFP-GO	0.74	0.67	0.73
FunApriori Prasad et al., (2017)	0.57	0.61	0.58
Chi square #1and2 Hishigaki et al., (2001)	0.13	0.12	0.12
Chi square #1 Hishigaki et al., (2001)	0.12	0.15	0.13
Neighborhood counting #1and2 Schwikowski et al., (2000)	0.21	0.25	0.18
Neighborhood counting #1 Schwikowski et al., (2000)	0.15	0.21	0.17
Fs-weight #1and2 Chua et al., (2006)	0.24	0.22	0.22
Fs-weight #1 Chua et al., (2006)	0.16	0.19	0.19
Nrc Moosavi et al., (2013)	0.25	0.24	0.22

The major limitation of the chi-square method is that it is suitable only for the denser part of the network. Thus, network sparseness may lead to the degradation of performance evaluation compared to the others. The inclusion of level-1 and level-2 neighbors increases the accuracy rates in all except chi-square #1 and FS-weight #1 (using only the first level).

The neighborhood counting method is simple but still lags more like NRC and FS-weight #1 and #2 (using both levels) as it fails to differentiate between them. Although NRC and FunApriori perform better than the other, they fall behind PFP-GO since it does not focus on eliminating non-essential proteins from the target neighborhood.

PFP-GO based on only PPI network and sequence is also tested against similar kinds of existing methodologies like NAIVE (Murphy, 2006) and BLAST (Mount, 2007) method as reported in (Piovesan et al., 2015), Multi-Label Protein Function Prediction (ML_PFP) (Saha, Prasad, et al., 2018) and DeepGO (Kulmanov et al., 2018). Table 4 highlights the entire scenario. Moreover, the prediction performance of PFP-GO has also been evaluated with INGA separately on GO terms: Cellular Components (CC), Molecular Functions (MF) and Biological Process (BP), highlighted in Table 5. F_MAX_ score for BP, MF and CC has also been taken into account, and the same is compared with other methods like NetGO 3.0 (You et al., 2019), DeepGOPlus (Kulmanov and Hoehndorf, 2020), BLAST (Mount, 2007) and NAÏVE (Murphy, 2006). The result is displayed in Table 6. None of these methods considers filtering or including various sequence-derived features like an aliphatic index, etc., because they fail to perform better than PFP-GO. Moreover, Moreover, DeepGO cannot predict protein functions with a sequence length >1,000, which is another snag. ML_PFP has used protein sequence and PPI network quite effectively but uses only edge weight as the only parameter for screening the non-reliable edges in PPIN. In contrast, PFP-GO uses 2-pass filtering and pruning approach.

**TABLE 4:** Performance analysis of PFP-GO with other methods based on PPI network and sequence.

Methodology	Precision	Recall	F-score
PFP-GO	0.52	0.64	0.56
Deep_GO Kulmanov et al., (2018)	0.48	0.49	0.48
BLAST Mount, (2007); Piovesan et al., (2015)	0.30	0.50	0.37
NAÏVE Murphy, (2006); Piovesan et al., (2015)	0.33	0.31	0.31

**TABLE 5:** Performance analysis of INGA and PFP-GO separately on CC, MF and BP.

	Precision			Recall			F-score		
Methodology	BP	MF	CC	BP	MF	CC	BP	MF	CC
PFP-GO	0.49	0.51	0.48	0.95	0.98	0.95	0.64	0.67	0.64
INGA Piovesan et al., (2015)	0.37	0.53	0.42	0.33	0.63	0.63	0.58	0.57	0.49

**TABLE 6:** Performance analysis of PFP-GO with other methods based on Fmax score.

Methodology	BP	MF	CC
PFP-GO	0.65	0.61	0.66
NetGO 3.0 You et al., (2019)	0.64	0.43	0.66
Deep_GO_Plus Kulmanov and Hoehndorf, (2020)	0.57	0.41	0.59
BLAST Mount, (2007); Piovesan et al., (2015)	0.63	0.31	0.56
NAÏVE Murphy, (2006); Piovesan et al., (2015)	0.4	0.23	0.54

To further validate the predicted and ranked Gene Ontology terms, we used the meta-network created in (Halder et al., 2020) and (Chiliński et al., 2021) to study the importance of some of the GO terms in the perspective of the 3D-structure of the genome. To include this assessment, we create a Gene-Gene association network following the ideas presented in the work of Chiliński et al. (2021); Halder et al. (2020). We derive the Genomic association network from the 3D chromatin structure. After we created the networks, we mapped the unknown test set to the network and found the level-1 and level-2 neighboring genes from each target node. We end up with a similar tree, as shown in Figure 5. However, here the nodes in the tree are genes. Then we map the genes to the GO terms from the leaf nodes and propagate them to the target node (Sengupta et al., 2018). Once all the GO terms are fetched, each of them is ranked by computing its enrichment in the PPI network using the *p* value with Fisher’s exact test (Piovesan et al., 2015). Top-ranked GO terms are finally allocated to the target protein. From the ranking, we obtained the top Gene ontology terms, which are displayed in Table 7.

**TABLE 7:** Top-ranked gene ontology terms selected from GGA validation.

Rank	Gene	GO-terms
1	ENSG00000123131	GO:0000049
2	ENSG00000123131	GO:0001731
3	ENSG00000123131	GO:0003743
4	ENSG00000130741	GO:0005576
5	ENSG00000130741	GO:0005634
6	ENSG00000130741	GO:0005783

## Conclusion

From Table 2, Table 3, and Table 4, it can be inferred that our method PFP-GO outperforms the other methodologies in the same dataset of humans in terms of precision, recall, and F-score values due to several reasons: 1) The target set of proteins are selected from high-ranking GO terms which implies the fact that only proteins having high connectivity are involved. 2) Pruning and double filtering of proteins are executed by eliminating Bridge, Shore, and Fjord proteins (non-essential proteins) which have not been taken into account by the other methods, which is the primary cause for the increase in false rates. 3) Besides consideration of every GO and non-GO term, prediction provides a proper equilibrium in the proposed methodology. 4) Moreover, PFP-GO combines prediction from three orthogonal sources, i.e., sequence-based predictor, domain interaction network-based predictor, and protein interaction network-based predictor, to predict the protein’s function. All these lead to the enhancement of our prediction accuracy.

Besides, it should be noted that PFP-GO can perform better whether it considers the PPI network alone, PPI network and sequence, or the combination of the trio: PPI network, sequence, and domain. It can also identify functionally active proteins which may be transmitted in identifying possible drug targets in the future (Anup Kumar Halder et al., 2018; Saha, Sengupta, et al., 2018). Recently our work has been limited to human-specific datasets, which can be extended further to other organisms. The PFP-GO software package and the complete source code are available in the public domain for noncommercial research use at https://sites.google.com/view/pfp-go/.

## Data Availability

The datasets presented in this study can be found in online repositories. The names of the repository/repositories and accession number(s) can be found in the article/supplementary material.
